# Long‐Term Efficacy of Restricted Quantitative Superior Placement Technique in Total Hip Arthroplasty for Severe Developmental Dysplasia of the Hip: An 8‐ to 10‐Year Follow‐Up of a Prospective Randomized Controlled Trial

**DOI:** 10.1111/os.70448

**Published:** 2026-11-05

**Authors:** Shuai Li, Zhijian Yang, Dong Jiang, Yu Xie, Xiaomin Lu, Xiaoyi Zhao, Minghui Gu, Zhiwen Li, Weiming Liao, Zhiqi Zhang

**Affiliations:** ^1^ Department of Joint Surgery The First Affiliated Hospital of Sun Yat‐sen University Guangzhou Guangdong China; ^2^ Guangdong Provincial Key Laboratory of Orthopedics and Traumatology, The First Affiliated Hospital of Sun Yat‐sen University Sun Yat‐sen University Guangzhou China; ^3^ Jieyang People's Hospital Jieyang Guangdong China

**Keywords:** developmental dysplasia of the hip, randomized controlled trial, restricted quantitative superior placement, total hip arthroplasty

## Abstract

**Objective:**

Total hip arthroplasty (THA) in patients with developmental dysplasia of the hip (DDH), particularly those with Crowe II or III dysplasia, is challenging because of abnormal acetabular morphology and deficient bone stock. This study aims to compare the long‐term outcomes of true anatomic versus restricted quantitative superior placement of the acetabular component.

**Methods:**

This prospective, patient‐ and assessor‐blinded randomized controlled trial was conducted from August 1, 2013, to July 31, 2024. Forty‐four hips were allocated 1:1 to true anatomic center placement or restricted quantitative superior placement. Restricted quantitative superior placement was defined as positioning the cup center no more than 15 mm superior to the true anatomic center. Harris Hip Score (HHS), 36‐Item Short Form Health Survey (SF‐36), complications, perioperative outcomes, and radiographic parameters were assessed. The mean final follow‐up was 8.94 years. Independent‐samples *t‐*test, Mann–Whitney U test, Chi‐square test, and Fisher's exact test were used for statistical analysis.

**Results:**

No statistically significant between‐group difference was observed in prosthetic complications. No revisions were recorded among the 36 hips that completed the 8–10‐year follow‐up. At the 8–10‐year follow‐up, the restricted quantitative superior placement group had a higher HHS than the true anatomic center group (93.70 ± 5.20 vs. 86.73 ± 11.62, *p* = 0.024); multiple imputation analysis showed a mean difference of 5.79 points (95% CI, 0.46–11.13; *p* = 0.034). Significant between‐group differences were also observed at 2 and 7 years. SF‐36 scores did not differ significantly between groups. Morselized bone grafting was required in 45.5% of the anatomic center group and 40.9% of the restricted quantitative superior center group. Structural grafting was used in 18.2% and 9.1%, respectively. Radiographic analysis showed higher center of rotation height and less limb lengthening in the restricted quantitative superior center group.

**Conclusions:**

Restricted quantitative superior placement within 15 mm above the true anatomic center was associated with higher long‐term HHS, with no significant difference in prosthetic complications and no revisions recorded among the 36 hips that completed the 8–10‐year follow‐up. It may represent a potential reconstructive option for patients with DDH.

**Trial Registration**: Chictr.org, ChiCTR‐TRC‐1404388; https://www.chictr.org.cn/showproj.html?proj=5182

## Graphical abstract

Overview of a randomized controlled trial comparing restricted quantitative superior placement with true anatomic center reconstruction in THA for DDH. Compared with anatomic placement, this technique reduced bone grafting, maintained adequate cup coverage, improved hip function, and achieved favorable long‐term outcomes without revision surgery.

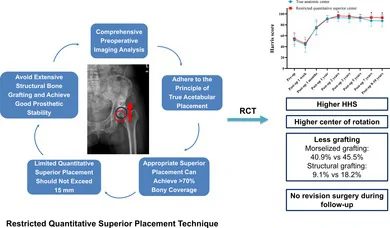

## 1 Introduction

Developmental dysplasia of the hip (DDH) with secondary hip osteoarthritis is a common indication for total hip arthroplasty (THA) [1]. However, anatomical abnormalities of the dysplastic acetabulum and deficient bone stock often lead to insufficient bone coverage of the true acetabulum [2], increasing surgical complexity and risk. For patients with severe hip dysplasia, reconstructing the true anatomic acetabulum can be achieved using bulk bone autograft [3], acetabular medial wall osteotomy [3, 4], metal augments [5] or acetabular component medialization [6]. However, these approaches inevitably increase surgical time and costs, elevate postoperative complication rates, and impose usage limitations [3, 7].

In this context, we proposed a restricted quantitative superior placement strategy, based on fundamental biomechanical studies demonstrating that superior placement of the hip joint rotation center by less than 15 mm does not significantly increase joint stress [8]. This defined a biomechanically acceptable range for superior positioning of the rotation center. Subsequently, in 2013, we initiated a prospective randomized controlled trial (RCT) to evaluate the clinical efficacy of this technique defined as restricted quantitative superior placement of the hip center of rotation by no more than 15 mm. In our previously reported 12‐month analysis, early clinical outcomes did not differ significantly between the two groups, and restricted superior quantitative placement restored limb length without routinely requiring bulk structural grafting [9].

Long‐term randomized evidence comparing these two reconstruction strategies remains limited. This study aimed to evaluate the 8–10‐year outcomes of cementless acetabular components implanted using a restricted quantitative superior placement technique in patients with Crowe Type I–III developmental dysplasia of the hip with more than 35% subluxation. Specifically, we investigated: (i) survival rate and prosthetic complications; (ii) long‐term hip function and patient‐reported health status; (iii) radiographic outcomes, as well as the requirement for acetabular bone grafting.

## 2 Materials and Methods

### 2.1 Study Design

This prospective, parallel‐group, patient‐ and assessor‐blinded RCT was approved by the Clinical Research Ethics Committee of our hospital (IRB No. 2013‐32) on February 28, 2013. The trial was registered at the Chinese Clinical Trial Registry (ChiCTR‐TRC‐14004388). The execution period was recorded as spanning from August 1, 2013, to July 31, 2024. Patient recruitment officially began on August 1, 2013, and the first patient was enrolled on November 13, 2013. This RCT was designed and reported in accordance with the Consolidated Standards of Reporting Trials (CONSORT) 2010 Statement.

### 2.2 Participants

Patients were eligible for inclusion if they met the following criteria: (1) diagnosed with DDH classified as Crowe Type I with more than 35% subluxation, or Crowe Type II or Type III, accompanied by secondary osteoarthritis requiring THA; (2) aged between 30 and 80 years old at the time of surgery; and (3) able to independently complete the questionnaires.

Exclusion criteria included: (1) high surgical risk for using either true acetabular placement or superior placement techniques; (2) Crowe Type I DDH with less than 35% subluxation; (3) Crowe Type IV DDH; (4) neurological disorders associated with muscle weakness (e.g., multiple sclerosis, Parkinson's disease, or history of cerebral infarction); (5) coagulation disorders or active infections; (6) pregnancy; (7) receipt of intra‐articular injections within the past 6 months; (8) undergoing THA for diagnoses unrelated to DDH (e.g., rheumatoid arthritis, lupus erythematosus, ankylosing spondylitis, or psoriatic arthritis); (9) cancer; and (10) inability to complete long‐term follow‐up.

All patients signed informed consent. Preoperative evaluations and questionnaires were completed following random group allocation. To minimize potential bias, patients were informed that this study aimed to compare two different THA techniques, without indicating superiority of either approach. CONSORT flowchart outlining patient recruitment is presented in Figure 1.

**FIGURE 1:**
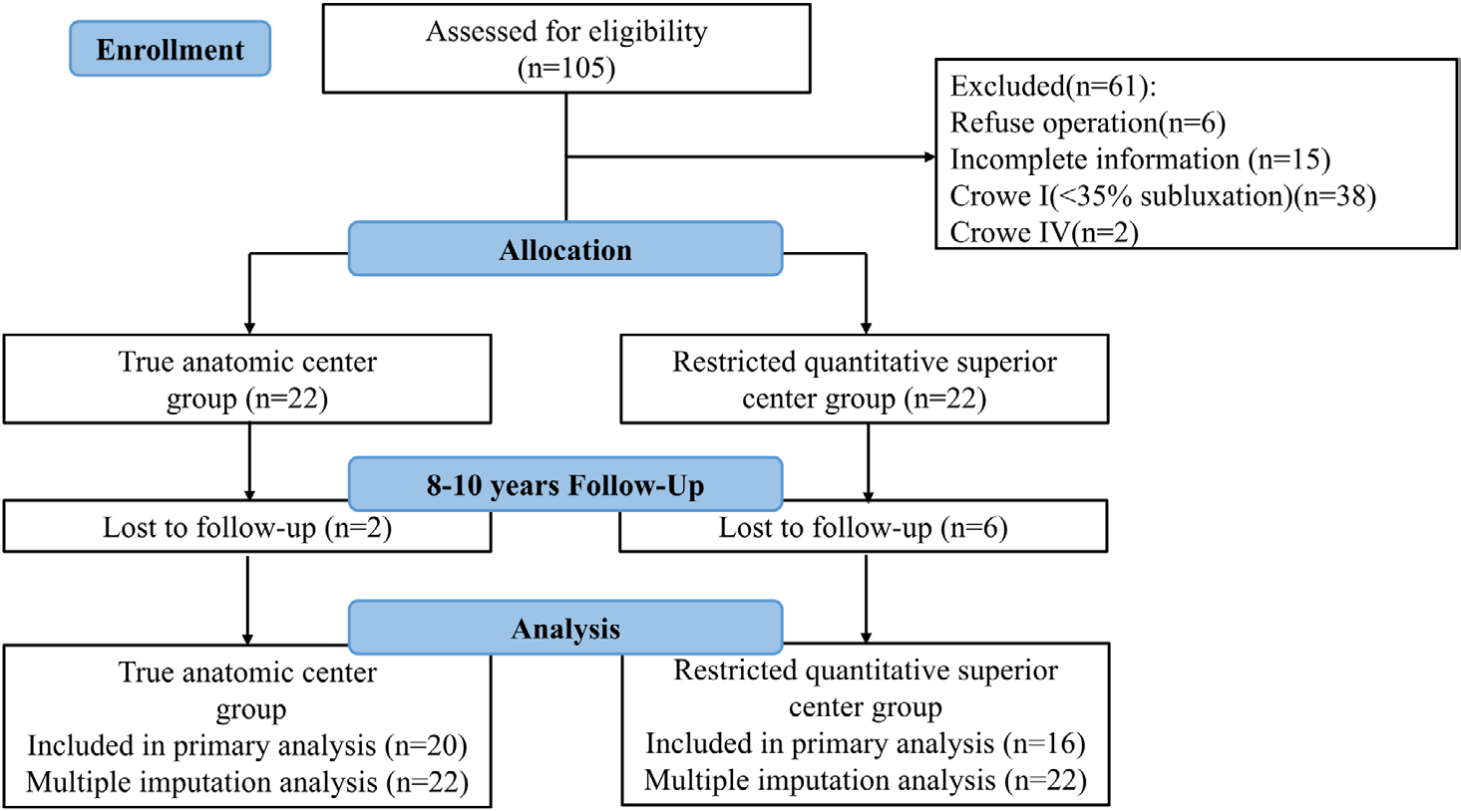
CONSORT flow diagram of patient enrollment, allocation, follow‐up, and analysis.

Group allocation was masked to independent analysts.

The sample size was calculated before enrollment using previous reports [10, 11] and our earlier work [8]. The 10‐year acetabular prosthesis survival rate for patients with DDH undergoing true anatomic center placement with bone grafting is approximately 67% (varying from 53% to 80%), due to complications such as graft resorption and collapse. In contrast, the superior placement technique, restricted to < 15 mm elevation, avoids bulk grafting and is not expected to decrease prosthesis longevity. Its assumed 10‐year survival rate is therefore estimated at 90%, similar to standard THA cases without acetabular deformity. Based on these assumptions, with a two‐sided significance level (*α*) of 5% and a statistical power (1 − *β*) of 85%, the minimum required sample size per group was calculated to be 20 hips. This accounts for detecting a statistically significant difference between the two groups. Accounting for an estimated 10% loss to follow‐up, we aimed to enroll at least 22 hips per group. Accordingly, the sample‐size calculation was based on the assumed between‐group difference in 10‐year acetabular prosthesis survival.

### 2.3 Randomization and Blinding

This study was designed as a parallel‐arm RCT with a 1:1 allocation ratio between the true anatomic center group and the restricted quantitative superior center group. Randomization was performed using a computer‐generated random number by the School of Public Health at our university, which was independent of the clinical team.

Patients were blinded to their group assignment. Surgeons were informed of the group allocation by a sealed opaque envelope immediately before surgery and were not involved in any postoperative follow‐up or outcome assessments. Postoperative evaluations were conducted by independent investigators who were blinded to group assignments.

### 2.4 Surgical Technique

Five experienced orthopawedic surgeons participated in the trial. To ensure consistency and minimize variability, all surgeons underwent a standardized training program on both the true anatomic center and restricted quantitative superior placement techniques prior to trial initiation. Surgical adherence to protocol was monitored throughout the study.

All surgical procedures were performed according to a previously published protocol [9]. The techniques for both the true anatomic center placement and the restricted quantitative superior center placement have been described in detail [9]. In brief, all surgeries were performed using a posterolateral approach without trochanteric osteotomy. Osteophytes at the bottom of the acetabulum were reamed to expose the native acetabular fossa, allowing identification of the true acetabular center. For patients in the true anatomic center group, the acetabulum was reamed line‐to‐line at an appropriate angle of abduction and anteversion. Any acetabular cysts were curetted, and the resulting defects were filled with morselized autograft. Structural autografts from the femoral head were used as needed to ensure at least 70% bone coverage of the component surface.

For patients in the restricted quantitative superior‐center group, patient‐specific preoperative planning was performed using anteroposterior (AP) radiographs and three‐dimensional computed tomography (CT) reconstructions (Figure 2). Virtual cup implantation was used to determine the feasible superior position of the center of rotation that was expected to provide at least 70% bone coverage while remaining no more than 15 mm above the true anatomic center. If satisfactory host‐bone coverage could not be achieved even at the 15‐mm superior limit, structural autografting using the resected femoral head was performed rather than placing the acetabular component further superiorly.

**FIGURE 2:**
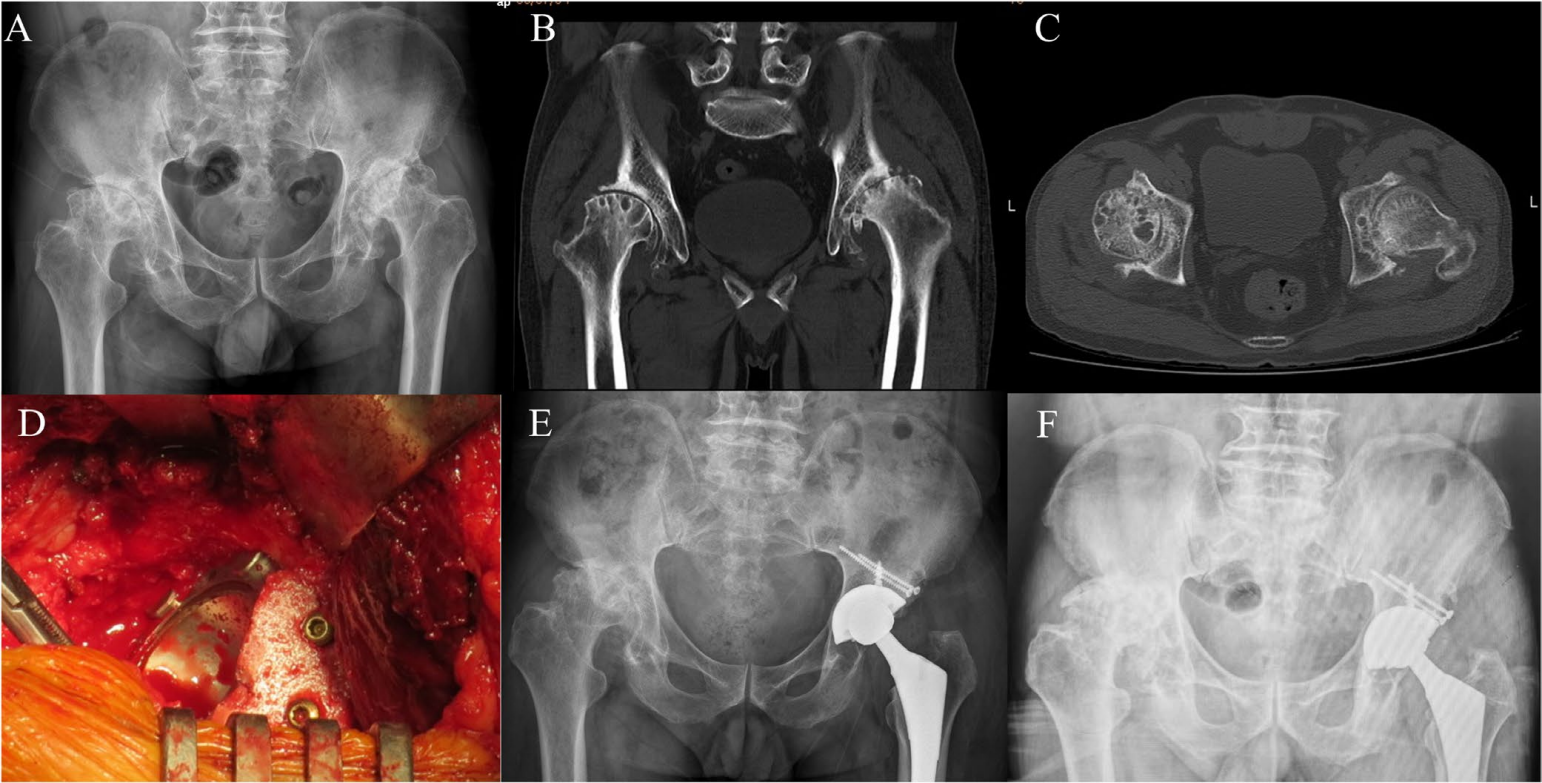
Preoperative and postoperative radiographic images of a 60‐year‐old male with Crowe II DDH with secondary osteoarthritis assigned to the restricted quantitative superior center group. (A) Preoperative X‐ray. (B and C) Preoperative CT images demonstrating bilateral hip developmental dysplasia. (D) Intra‐acetabulum structural bone autograft using the femoral head in the left THA. (E) Immediate postoperative in‐hospital X‐ray showing the acetabular component was positioned 8.7 mm superior to the true anatomic center. Satisfactory bone coverage of acetabular component was achieved. (F) The 9‐year follow‐up radiograph showed no evidence of component loosening or obvious liner wear.

Intraoperatively, the transverse acetabular ligament and superior acetabular rim were used as anatomical landmarks, whereas the teardrop and interteardrop line were used as radiographic references under fluoroscopy. The acetabulum was sequentially reamed at the planned inclination and anteversion. Trial cup placement was adjusted to reproduce the patient‐specific position determined during preoperative planning and to obtain adequate host‐bone coverage and primary stability. Intraoperative fluoroscopy was used when necessary to verify cup height, inclination, and anteversion and to confirm that superior displacement did not exceed 15 mm.

All patients received cementless acetabular and femoral components with ceramic femoral heads and polyethylene liners. The implants were manufactured by DePuy Orthopaedics (Warsaw, IN, USA), Smith and Nephew (Memphis, TN, USA), or Biomet (Warsaw, IN, USA). Further details regarding the prosthetic components and their distribution are provided in Supplementary Table 1.

### 2.5 Perioperative Management

All patients received perioperative care according to a standardized management protocol described in previous publications [6, 9]. All patients received the same perioperative care pathway, irrespective of treatment allocation. Perioperative antibiotics were administered for infection prophylaxis, and low‐molecular‐weight heparin was used for prevention of deep‐vein thrombosis. Postoperative pain was managed with analgesic medication according to the routine clinical protocol. On the first postoperative day, patients began supervised isometric and isotonic exercises involving the muscles of the operated lower limb. Assisted ambulation with a walker or crutches was initiated on the second postoperative day. During the early rehabilitation period, hip flexion was restricted to 90°, and adduction beyond the neutral position was prohibited. The suction drain was generally removed approximately 48 h after surgery. Patients were discharged after routine postoperative assessment, and skin sutures were removed on postoperative Day 14.

### 2.6 Outcome Measures

The primary endpoint was prosthetic revision‐free survival. Postoperative complications included radiographic aseptic loosening (defined using Engh' criteria [12], with at least one radiographic sign required), periprosthetic fractures, dislocations, periprosthetic joint infection (PJI), and sciatic nerve palsy. Follow‐up time was calculated from the date of the index total hip arthroplasty, which was defined as time zero, to the first revision or removal of the component for any reason. Hips that had not undergone revision were censored at the date of the last available clinical or radiographic assessment confirming that the component remained in situ, or at the date of the final verified follow‐up, whichever was applicable.

The prespecified clinical outcomes assessed during follow‐up included the Harris Hip Score (HHS), the 36‐Item Short Form Survey (SF‐36). HHS and SF‐36 results were primarily analyzed using observed data from patients who completed the corresponding assessments. Multiple imputation (MI) was additionally performed as a sensitivity analysis to assess the potential influence of missing participant data (MPD). Assessments were conducted at 1 week, 3 months, 1 year, 2 years, 3 years, 5 years, 7 years, and at the most recent follow‐up (mean 8.94 years postoperatively).

The HHS is a widely used tool for evaluating hip function with a total score of 100 points across four domains: pain, function, deformity, and range of motion. Higher scores indicate better hip function [13]. The SF‐36 includes 36 items covering two domains, physical and mental health, with scores ranging from 0 to 100. Higher scores reflect a better general health status [14].

Secondary outcomes included in‐hospital observations and radiographic evaluation. In‐hospital observations included length of stay (LOS), total hospital cost, operative time, intraoperative blood loss, blood transfusion, intraoperative use of bone grafts. Radiographic evaluations were performed using standardized AP pelvic radiographs and lateral pelvic radiographs. Mimics research software (version 17.0, Materialise, Leuven, Belgium) was used to measure the following parameters: vertical distance from center of rotation to interteardrop line (VD_cor_), vertical distance from tip of greater trochanter to interteardrop line (VD_gt_), femoral offset (FO), global femoral offset (GFO), leg length discrepancy (LLD), bone coverage index (BCI), cup anteversion, cup inclination. A schematic diagram of the radiographic measurement method is provided in Figure 3. Radiographic parameters were defined as follows:

1. *VD*_*cor*_: Vertical distance from the center of rotation to the interteardrop line. The preoperative center of rotation was identified using Ranawat's triangle method [15].
2. *VD*_*gt*_: Vertical distance from the tip of the greater trochanter to the interteardrop line.
3. *Sharp angle (deg)*: The angle between the horizontal teardrop line and a line through the inferior teardrop point and the lateral rim of the acetabulum [16].
4. *Acetabular depth‐to‐width ratio (%)*: The ratio of the depth of the acetabulum to the distance between the inferior teardrop point and the lateral acetabular rim [16].
5. *FO*: Distance from the center of rotation to the femoral shaft axis, using the method by Lecerf [17].
6. *GFO*: the sum of FO and horizontal distance [17]. The horizontal distance from the perpendicular point of the center of rotation on the interteardrop line to the bottom of the teardrop.
7. *LLD*: Difference between the vertical distances from the apexs of lesser trochanters to the interteardrop line on the non‐surgical side and surgical side [17].
8. *BCI*: Percentage of horizontal cup coverage, measured as the ratio of the distance from the medial edge of the cup to the lateral edge of bone coverage to the total cup width, based on the method described by Takao [18].
9. *Cup anteversion*: Measured using the method described by Woo [19].
10. *Cup inclination*: also called acetabular abduction. Defined as the angle between the interteardrop line and the tangential line along the acetabular component opening.

**FIGURE 3:**
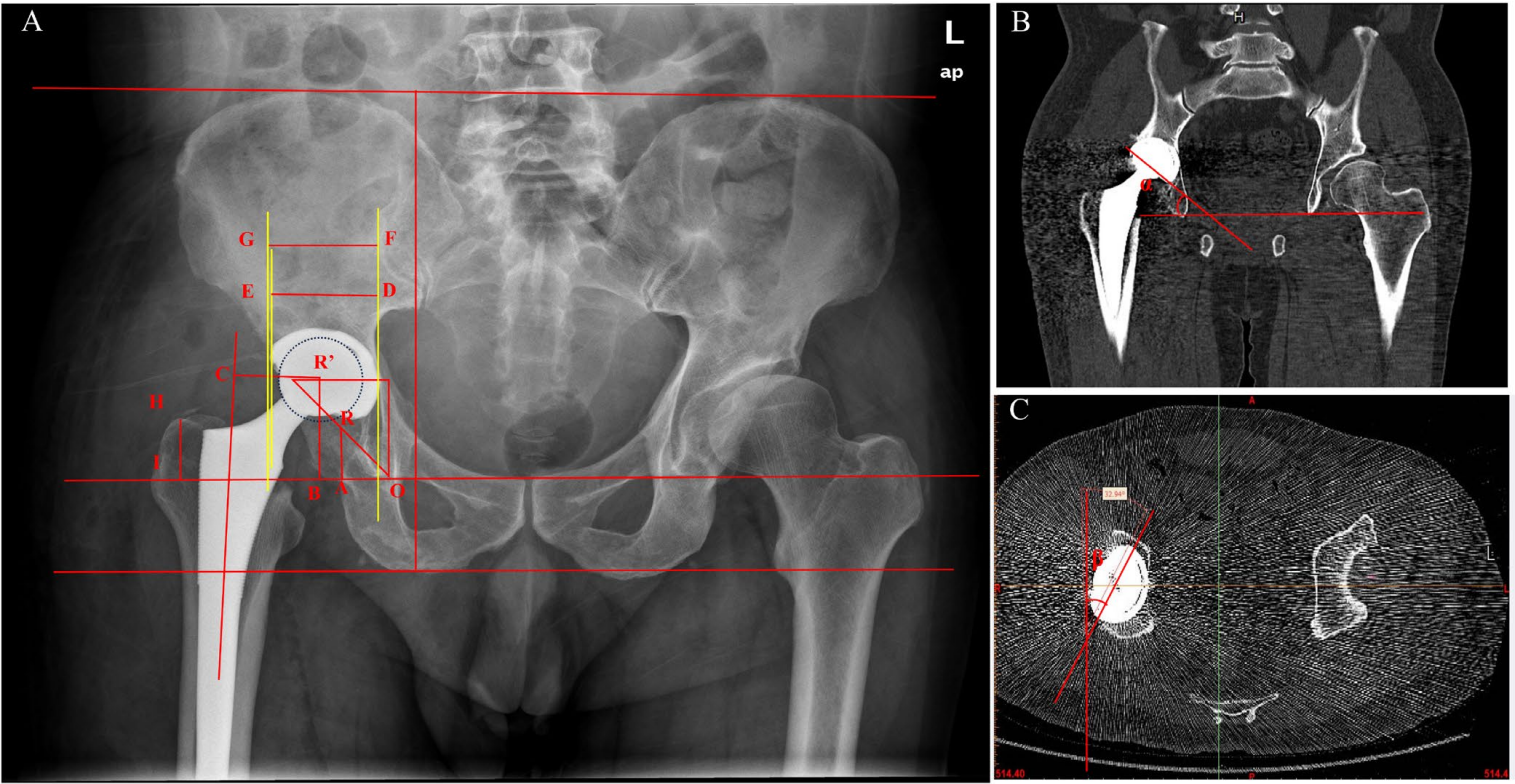
Radiographic evaluation based on anteroposterior (AP) pelvic radiographs. (A) RA: Vertical distance from the center of rotation to the interteardrop line, VD_cor_. R is the rotation center determined by Ranawat's triangle method. R′B: Vertical distance from the center of postoperative rotation to the interteardrop line. R′B − RA: Superior placement distance. HI: Vertical distance from the tip of the greater trochanter to the interteardrop line, VD_gt_. R′C: Distance from the center of rotation to the femoral shaft axis, *FO*. R′C + BO: Global femoral offset, GFO, the sum of FO and horizontal distance. BCI: Bone coverage index, DE/FG, the ratio of the distance from the medial edge of the cup to the lateral edge of bone coverage to the total cup width. (B) *α* angle: Cup inclination. (C) *β* angle: Cup anteversion.

All measurements were performed independently by two chief physicians specializing in hip. Interobserver reliability for continuous variables was assessed using the intraclass correlation coefficient based on a two‐way random‐effects model for absolute agreement [ICC(2,1)], with 95% confidence intervals. After the reliability analysis, the mean of the two measurements was used for the primary radiographic analysis.

### 2.7 Statistical Analysis

Primary analyses of HHS and SF‐36 were based on observed data, including all patients with available outcome data at the corresponding follow‐up assessment. To evaluate the robustness of the primary results to missing outcome data, multiple imputation was performed as a sensitivity analysis under the missing‐at‐random (MAR) assumption. Twenty imputed datasets were generated using multiple imputation by chained equations (MICE), with linear regression models used to impute the continuous HHS and SF‐36 scores. The imputation models included treatment group, age, sex, BMI, the corresponding baseline outcome score, and available longitudinal HHS and SF‐36 measurements throughout follow‐up. Each imputed dataset was analyzed using the same between‐group comparison approach as that used in the primary observed‐data analysis, and estimates were pooled across the 20 datasets using Rubin's rules [20].

For continuous variables, normality was assessed using the Kolmogorov–Smirnov (K–S) and Shapiro–Wilk (S–W) tests. Normally distributed variables were presented as mean ± standard deviation (SD) and were compared using the independent‐sample *t*‐test. Nonnormally distributed variables were analyzed using the Mann–Whitney U test. Categorical variables were presented as number of cases and percentage. The Chi‐square test and Fisher's exact test were used for comparisons of frequency distribution of categorical variables between independent groups. Statistical analysis was conducted using the SPSS software (Version 26.0, IBM, USA) with statistical significance set at *p* < 0.05. Complete‐case analyses were reported as the primary analyses for HHS/SF‐36, and MI was performed as a sensitivity analysis.

## 3 Results

### 3.1 Patient Characteristics

Initially, 105 hips with DDH were recruited for the study. After exclusion, 44 hips were enrolled in the study and randomized. All enrolled patients completed the 2‐year postoperative follow‐up.

At the final follow‐up (mean duration: 8.94 years), 36 hips completed both in‐hospital evaluation and follow‐up case report forms. The number of patients available at each follow‐up time point is presented in Figure 4, and their baseline characteristics are provided in Supplementary Table 2. The overall loss‐to‐follow‐up rate was 18.2%. Baseline demographic and clinical characteristics of patients are summarized in Table 1. There were no statistically significant differences between groups in any of the baseline parameters.

**FIGURE 4:**
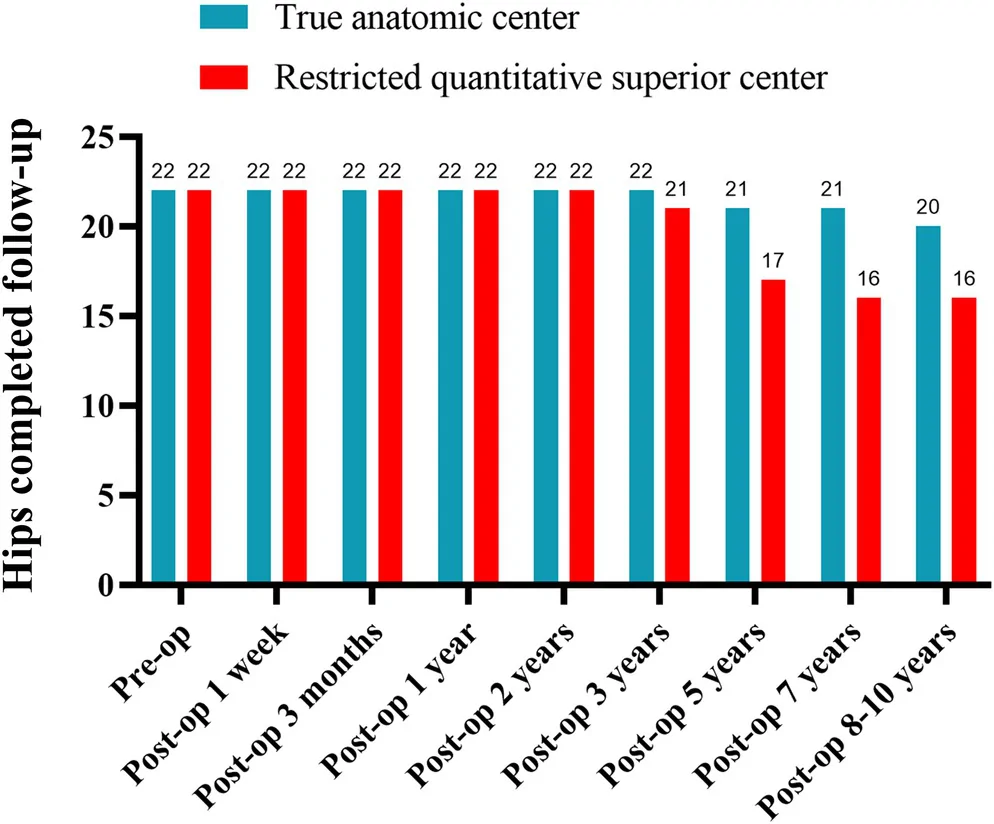
Number of hips completing follow‐up at each time point in both groups.

**TABLE 1:** Baseline demographic, clinical, and preoperative radiographic characteristics of the patients.

	True anatomic center (*n* = 22)	Restricted quantitative superior center (*n* = 22)	Statistics	*p*
Age (years)	58.73 ± 11.06	57.95 ± 8.53	0.259	0.797
Height (cm)	157.55 ± 7.85	160.82 ± 6.32	−1.522	0.136
Weight (kg)	61.73 ± 10.95	62.18 ± 14.35	−0.118	0.907
BMI (kg/m^2^)	24.91 ± 4.48	23.95 ± 4.91	0.682	0.499
Sex, no. of hips (%)			0.419	0.517
Female	16 (72.7)	14 (63.6)		
Male	6 (27.3)	8 (36.4)		
Side for THA, no. of hips (%)			0.820	0.365
Left	13 (59.1)	10 (45.5)		
Right	9 (40.9)	12 (54.5)		
Classification, no. of hips				
Crowe I	16	14		
Subluxation (%)	40.14 ± 4.34	40.64 ± 3.61	−0.343	0.734
Crowe II	4	7		
Subluxation (%)	60.5 ± 9.12	60.03 ± 5.48	0.073	0.943
Crowe III	2	1		
Subluxation (%)	86.89 ± 13.92	80.21		0.762
Average subluxation (%)	48.06 ± 15.96	48.61 ± 12.24	−0.127	0.899
Preoperative HHS	49.64 ± 12.24	51.13 ± 9.75	−0.461	0.646
Preoperation SF‐36	59.23 ± 7.06	60.82 ± 6.71	−0.766	0.448
Preoperative LLD (mm)	9.95 ± 19.31	8.81 ± 9.81	0.245	0.808
Preoperative VD_cor_ (mm)	22.41 ± 1.09	22.51 ± 1.60	−0.245	0.808
Sharp angle (deg)	46.59 ± 5.77	47.32 ± 4.54	−0.465	0.645
Acetabular depth‐to‐width ratio (%)	25.12 ± 4.92	26.06 ± 5.02	−0.633	0.530

*Note:* Continuous variables are reported as mean ± standard deviation. Categorical variables are presented as number (%). Comparisons between groups were performed using *t*‐test or Mann–Whitney U test, as appropriate. Chi‐squared (*χ* ^2^) test is used to compare categorical variables.

Abbreviations: BMI, body mass index; HHS, Harris Hip Score; LLD, limb length discrepancy; THA, total hip arthroplasty; VD_cor_, vertical distance from center of rotation to interteardrop line.

### 3.2 Complications

With a minimum 8‐year follow‐up, one patient in the true anatomic center group (5.0%) and one patient in the restricted quantitative superior center group (6.3%) exhibited radiographic signs of aseptic loosening. Neither patient reported significant hip pain. Additionally, one dislocation occurred in the true anatomic center group and was successfully treated by closed reduction. No cases of periprosthetic fracture, periprosthetic joint infection, or sciatic nerve palsy were observed in either group (Table 2).

**TABLE 2:** Postoperative complications observed during follow‐up.

	True anatomic center (*n* = 20)	Restricted quantitative superior center (*n* = 16)	Statistics	*p*
Radiographic loosening, no. of hips (%)	1 (5.0)	1 (6.3)	—	1.000
Acetabular component	1 (5.0)	1 (6.3)	—	1.000
Femoral component	0 (0.0)	0 (0.0)	—	—
With pain	0 (0.0)	0 (0.0)	—	—
Periprosthetic fractures, no. of hips (%)	0 (0.0)	0 (0.0)	—	—
Dislocations, no. of hips (%)	1 (5.0)	0 (0.0)	—	1.000
PJI, no. of hips (%)	0 (0.0)	0 (0.0)	—	—
Revision rate (%)	0 (0.0)	0 (0.0)	—	—

*Note:* Categorical variables were compared using Fisher's exact test.

Abbreviation: PJI, periprosthetic joint infection.

### 3.3 HHS

HHS values are summarized in Table 3 and Figure 5. Both groups showed substantial postoperative improvement. Compared with the true anatomic center group, the restricted quantitative superior center group showed significantly higher HHS values at 2 years, 7 years, and 8–10 years postoperatively (*p* = 0.045, *p* = 0.021, *p* = 0.024, respectively). Sensitivity analysis using multiple imputation yielded consistent findings. The between‐group differences remained significant at 7 years (mean difference, −5.75 points; 95% CI, −10.90, −0.61, *p* = 0.030) and 8–10 years (mean difference, −5.79 points; 95% CI, −11.13, −0.46; *p* = **0.034**) whereas no significant differences were observed at 3 or 5 years (Table 4).

**TABLE 3:** Harris Hip Score (HHS) of patients during follow‐up.

	True anatomic center	Restricted quantitative superior center	Statistics	*p*
Pre‐operation	49.64 ± 12.24 (*n* = 22)	51.13 ± 9.75 (*n* = 22)	−0.461	0.646
1 week	43.44 ± 13.78 (*n* = 22)	45.71 ± 7.78 (*n* = 22)	−0.672	0.505
3 months	75.56 ± 14.19 (*n* = 22)	74.52 ± 12.65 (*n* = 22)	0.256	0.799
1 year	91.56 ± 5.19 (*n* = 22)	90.84 ± 6.15 (*n* = 22)	0.419	0.677
2 years	93.12 ± 6.74 (*n* = 22)	96.42 ± 2.95 (*n* = 22)	−2.100	**0.045**
3 years	92.69 ± 7.98 (*n* = 22)	96.02 ± 4.91 (*n* = 21)	−1.639	0.109
5 years	92.28 ± 7.12 (*n* = 21)	93.43 ± 5.86 (*n* = 17)	−0.536	0.595
7 years	87.36 ± 10.94 (*n* = 21)	93.96 ± 5.21 (*n* = 16)	−2.428	**0.021**
8–10 years	86.73 ± 11.62 (*n* = 20)	93.70 ± 5.20 (*n* = 16)	−2.398	**0.024**

*Note:* Continuous variables are reported as mean ± standard deviation. Comparisons between groups were performed using *t*‐test or Mann–Whitney U test. Bold values indicate statistically significant differences (*p* < 0.05).

**FIGURE 5:**
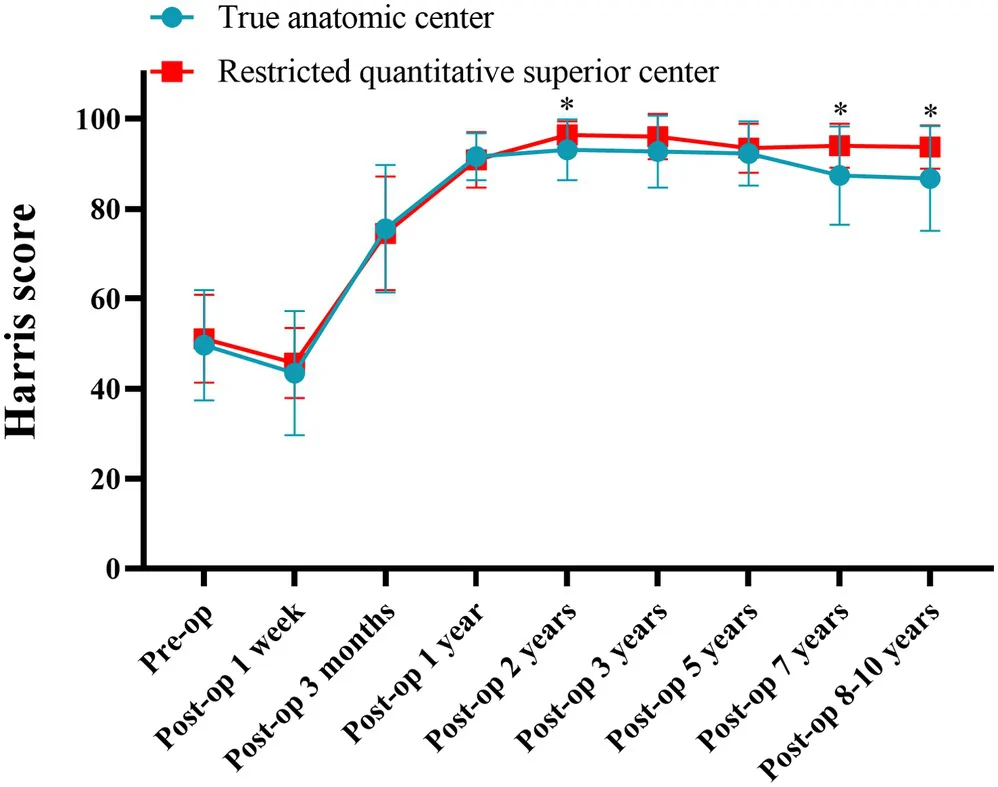
Comparison of Harris Hip score (HHS) between the two groups. * indicates *p* < 0.05.

**TABLE 4:** Comparison of complete‐case and multiple imputation sensitivity analyses for HHS and SF‐36 outcomes.

Outcome	Complete‐case analysis			Multiple imputation for MPD		
	Control vs. intervention			Control vs. intervention		
	Mean difference	95% CI	*p*	Mean difference	95% CI	*p*
Postoperative HHS						
3 years	−3.33	(−7.43, 0.77)	0.109	−3.03	(−7.11, 1.04)	0.140
5 years	−1.15	(−5.51, 3.20)	0.595	−0.55	(−4.36, 3.27)	0.774
7 years	**−6.60** ^a^	(−12.15, −1.05)	**0.021**	**−5.75** ^a^	(−10.90, −0.61)	**0.030**
8–10 years	**−6.97** ^a^	(−12.92, −1.01)	**0.024**	**−5.79** ^a^	(−11.13, −0.46)	**0.034**
Postoperative SF‐36 scores						
3 years	−1.65	(−4.19, 0.90)	0.198	−1.6	(−4.08, 0.88)	0.200
5 years	−0.99	(−3.79, 1.80)	0.476	−1.22	(−3.66, 1.24)	0.332
7 years	−1.79	(−4.17, 0.58)	0.134	−1.53	(−3.53, 0.46)	0.129
8–10 years	−1.38	(−3.78, 1.03)	0.264	−1.18	(−3.20, 0.85)	0.247

*Note:* Control: True anatomic center. Intervention: Restricted quantitative superior center. Differences between randomized groups for continuous outcome variables were analyzed using the *t*‐test. Bold values indicate statistically significant differences (*p* < 0.05).

Abbreviations: CI, Confidence interval; HHS, Harris Hip Score; MPD, missing participant data; SF‐36, 36‐Item Short Form Health Survey.

^a^ Statistical significance between groups.

An exploratory subgroup analysis was conducted among patients with bilateral DDH who underwent unilateral THA. The restricted quantitative superior center group showed higher HHS values at 2 years, 3 years, 7 years, and 8–10 years postoperatively than the true anatomic center group (*p* = 0.012, *p* = 0.020, *p* = 0.042, *p* = 0.010, respectively) (Table 5 and Figure 6). However, given the small subgroup size, these findings should be interpreted as exploratory and hypothesis‐generating rather than confirmatory.

**TABLE 5:** Harris Hip Score (HHS) of patients with bilateral DDH undergoing unilateral total hip arthroplasty during follow‐up.

	True anatomic center	Restricted quantitative superior center	Statistics	*p*
Pre‐operation	53.61 ± 10.47 (*n* = 12)	54.09 ± 10.62 (*n* = 9)	−0.101	0.920
1 week	42.46 ± 12.11 (*n* = 12)	46.89 ± 7.51 (*n* = 9)	−0.963	0.348
3 months	75.57 ± 15.65 (*n* = 12)	74.89 ± 12.10 (*n* = 9)	0.110	0.914
1 year	91.55 ± 6.12 (*n* = 12)	90.16 ± 5.16 (*n* = 9)	0.443	0.663
2 years	90.79 ± 7.94 (*n* = 12)	97.90 ± 2.75 (*n* = 9)	−2.977	**0.012**
3 years	90.54 ± 9.45 (*n* = 12)	98.08 ± 2.47 (*n* = 9)	−2.647	**0.020**
5 years	90.54 ± 8.66 (*n* = 12)	94.57 ± 5.72 (*n* = 9)	−1.205	0.243
7 years	86.39 ± 12.68 (*n* = 12)	95.23 ± 4.50 (*n* = 9)	−1.986	**0.042**
8–10 years	84.27 ± 13.40 (*n* = 12)	96.31 ± 2.85 (*n* = 9)	−3.020	**0.010**

*Note:* Continuous variables are reported as mean ± standard deviation. Comparisons between groups were performed using *t*‐test or Mann–Whitney *U* test, as appropriate. Bold values indicate statistically significant differences (*p* < 0.05).

Abbreviation: THA, total hip arthroplasty.

**FIGURE 6:**
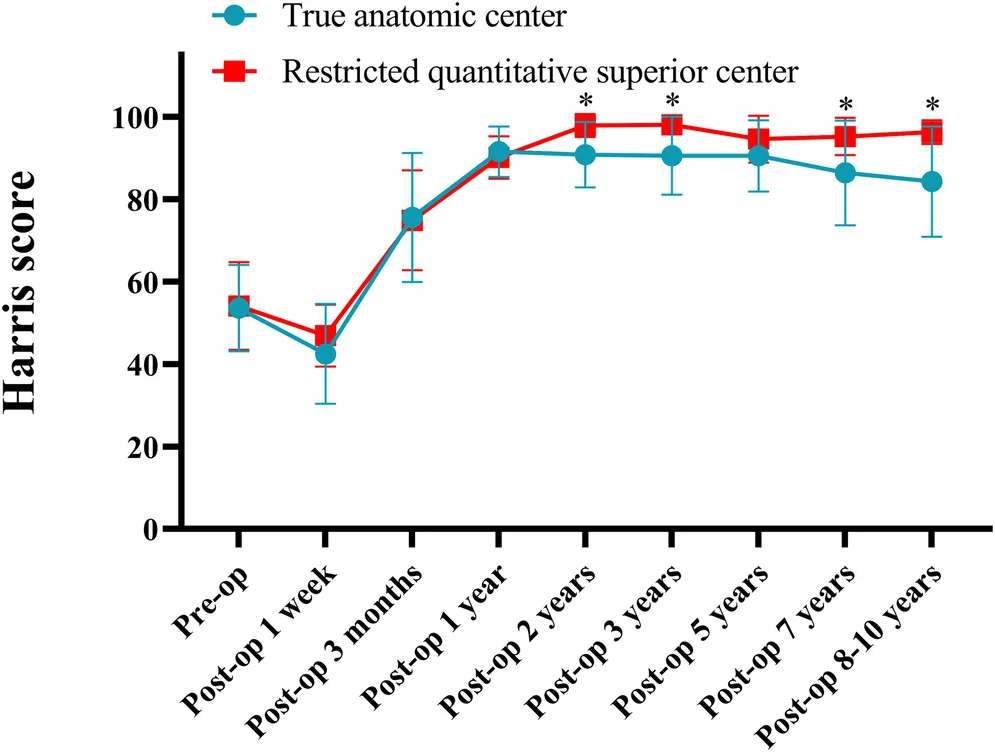
Comparison of Harris Hip Score (HHS) of patients with bilateral DDH undergoing unilateral THA between the two groups. * indicates *p* < 0.05. THA, total hip arthroplasty.

### 3.4 SF‐36 Scores

The SF‐36 scores for both groups are shown in Table 6 and Figure 7. Scores improved in both groups, with no statistically significant between‐group difference at any follow‐up point. Sensitivity analysis based on multiple imputation produced results consistent with those of the primary analysis (Table 4).

**TABLE 6:** SF‐36 scores of patients during follow‐up.

	True anatomic center	Restricted quantitative superior center	Statistics	*p*
Pre‐operation	59.23 ± 7.06 (*n* = 22)	60.82 ± 6.71 (*n* = 22)	−0.766	0.448
1 week	62.93 ± 5.58 (*n* = 22)	63.27 ± 4.84 (*n* = 22)	−0.210	0.835
3 months	71.05 ± 6.94 (*n* = 22)	69.63 ± 4.31 (*n* = 22)	1.020	0.314
1 year	75.21 ± 3.68 (*n* = 22)	76.04 ± 3.85 (*n* = 22)	−0.730	0.470
2 years	75.13 ± 4.83 (*n* = 22)	76.57 ± 5.00 (*n* = 22)	−0.973	0.336
3 years	74.83 ± 4.45 (*n* = 22)	76.48 ± 3.76 (*n* = 21)	−1.308	0.198
5 years	75.89 ± 4.81 (*n* = 21)	76.88 ± 3.37 (*n* = 17)	−0.720	0.476
7 years	75.54 ± 3.71 (*n* = 21)	77.34 ± 3.26 (*n* = 16)	−1.535	0.134
8–10 years	75.50 ± 3.89 (*n* = 20)	76.88 ± 3.22 (*n* = 16)	−1.136	0.264

*Note:* Continuous variables are reported as mean ± standard deviation. Comparisons between groups were performed using *t*‐test or Mann–Whitney *U* test, as appropriate.

**FIGURE 7:**
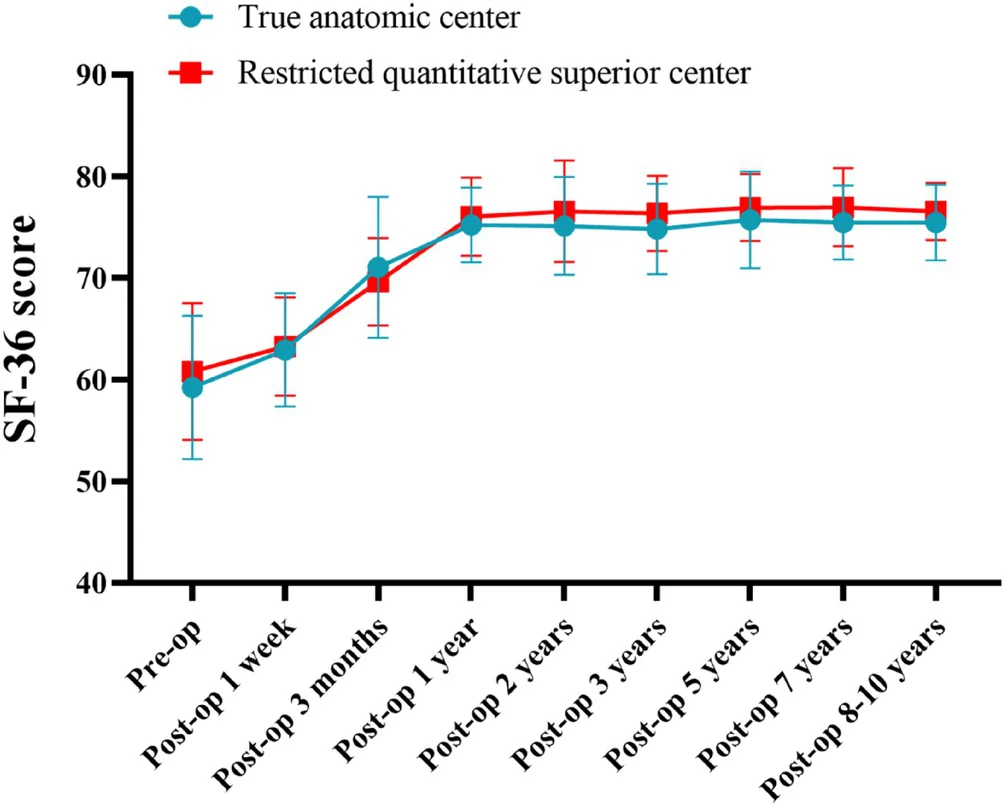
Comparison of SF‐36 scores of patients between the two groups.

### 3.5 In‐Hospital Observations

In‐hospital data are summarized in Table 7. There were no statistically significant differences between the two groups in LOS, total hospital cost, operative time, intraoperative blood loss, or blood transfusion volume.

**TABLE 7:** In‐hospital observations of patients.

	True anatomic center (*n* = 22)	Restricted quantitative superior center (*n* = 22)	Statistics	*p*
LOS (days)	16.64 ± 6.94	16.68 ± 4.77	−0.025	0.980
Total hospital cost (¥)	58960.76 ± 15573.00	62088.22 ± 13959.38	−0.701	0.487
Operative time (min)	162.05 ± 61.31	142.55 ± 78.07	0.921	0.362
Intraoperative blood loss (mL)	413.64 ± 241.63	515.91 ± 321.97	−1.192	0.240
Blood transfusion (mL)	168.18 ± 295.02	90.91 ± 300.65	0.860	0.394
Bone graft in‐use, no. of hips (%)				
Morselized bone graft	10 (45.5)	9 (40.9)	0.093	0.761
Structural bone graft	4 (18.2)	2 (9.1)		0.664

*Note:* Continuous variables are reported as mean ± standard deviation. Categorical variables are presented as number (%). Comparisons between groups were performed using *t*‐test or Mann–Whitney U test, as appropriate. The Chi‐squared (*χ* ^2^) test and Fisher's exact test were used to compare categorical variables.

Abbreviation: LOS, length of stay.

Morselized bone grafting was performed in 45.5% of patients (10/22) in the true anatomic center group and 40.9% (9/22) in the restricted quantitative superior center group. Structural bone grafting was required in 18.2% (4/22) and 9.1% (2/22) of patients in the respective groups. There were no significant differences in the number or type of bone grafts between the two groups.

All patients underwent successful operations without intraoperative complications. No in‐hospital complications, such as infection, deep vein thrombosis of the lower extremity, nerve injury, fracture, or dislocation, were observed in either group.

### 3.6 Radiographic Outcomes

Radiographic evaluations of prosthesis positioning are summarized in Table 8. A significant difference of the superior placement distance was observed between the true anatomic center group and the restricted quantitative superior center group (−0.14 ± 3.16 mm vs. 9.15 ± 3.01 mm, *p* < 0.001). Besides, a significant difference of postoperative VD_cor_ was observed between the two groups (22.27 ± 3.13 mm vs. 31.66 ± 3.65 mm, *p* < 0.001). There were no statistically significant differences in postoperative VD_gt_, FO, GFO, BCI, LLD, cup anteversion, and cup inclination between the two groups. However, limb lengthening was significantly greater in the true anatomic center group compared to the restricted quantitative superior center group (11.85 ± 7.41 mm vs. 5.56 ± 3.14 mm, *p* = 0.001). Interobserver ICCs for radiographic measurements ranged from 0.840 to 0.997. The reliability results are presented in Supplementary Table 3.

**TABLE 8:** Comparison of postoperative radiographic parameters between groups.

	True anatomic center (*n* = 22)	Restricted quantitative superior center (*n* = 22)	Statistics	*p*
Superior placement distance (mm)	−0.14 ± 3.16	9.15 ± 3.01	−9.981	**< 0.001**
VD_cor_ (mm)	22.27 ± 3.13	31.66 ± 3.65	−9.146	**< 0.001**
VD_gt_ (mm)	23.79 ± 10.36	27.17 ± 7.67	−1.231	0.225
FO (mm)	31.15 ± 4.76	34.32 ± 6.59	−1.833	0.075
GFO (mm)	58.55 ± 4.79	61.97 ± 7.52	−1.797	0.081
Postoperative LLD (mm)	−1.90 ± 17.85	3.26 ± 8.90	−1.213	0.234
BCI (%)	92.67 ± 5.43	94.88 ± 5.78	−1.332	0.190
Limb lengthening (mm)	11.85 ± 7.41	5.56 ± 3.14	3.662	**0.001**
Cup anteversion (deg)	23.78 ± 8.51	23.83 ± 10.88	−0.019	0.985
Cup inclination (deg)	45.83 ± 8.75	44.43 ± 6.66	0.594	0.556

*Note:* Continuous variables are reported as mean ± standard deviation. Comparisons between groups were performed using *t*‐test or Mann–Whitney *U* test. Bold values indicate statistically significant differences (*p* < 0.05).

Abbreviations: BCI, bone coverage index; FO, femoral offset; GFO, global femoral offset; LLD, leg length discrepancy; VD_cor_, vertical distance from center of rotation to interteardrop line; VD_gt_, vertical distance from tip of greater trochanter to interteardrop line.

## 4 Discussion

To our knowledge, this is the first RCT to evaluate the long‐term outcome of superior displacement of the hip center of rotation in total hip arthroplasty (THA) for patients with dysplasia of the hip (DDH). In this trial, the acetabular component was placed at a restricted quantitative superior position (averaging 9.15 ± 3.01 mm above the anatomic center) within the defined biomechanically safe zone of < 15 mm. At a mean follow‐up of 8.94 years, no revisions were recorded among the 36 hips that completed the 8–10 year follow‐up, and no statistically significant between‐group differences were observed in prosthetic complications. The restricted quantitative superior placement group had a significantly higher HHS at multiple follow‐up intervals (2, 7, and 8–10 years), whereas no statistically significant between‐group differences were observed in SF‐36 scores, BCI, FO, GFO, postoperative LLD, cup orientation, or acetabular bone‐grafting requirements.

### 4.1 Survival Rate and Prosthetic Complications

Acetabular reconstruction in DDH is challenging because marked anatomic deformity and superolateral bone deficiency increase surgical complexity, and superior hip‐center placement can improve host‐bone coverage by utilizing more favorable bone stock [3, 4, 21]. However, concerns remain that excessive superior displacement may alter hip biomechanics and increase the risks of polyethylene wear, aseptic loosening, and dislocation [22, 23, 24, 25]. In our study, the postoperative VD_cor_ in the restricted quantitative superior center group was 31.66 mm, yet no dislocation occurred in the restricted quantitative superior center group, and the single dislocation in the true anatomic center group was treated successfully by closed reduction and no revisions were recorded in either group during a minimum follow‐up of 8 years. Previous clinical series have reported long‐term cup survivorship of approximately 90%–97% after high hip‐center reconstruction at 12–15 years [22, 23, 26]. Chen et al. reported 5‐year survivorship rates of 90.3% for revision for any reason and 93.3% for aseptic loosening, despite a mean vertical cup position of 38.1 mm [27]. These findings suggest that superior placement can achieve durable fixation, but that the magnitude of elevation may remain clinically relevant. Our strategy limited superior displacement to within 15 mm, with maintenance of adequate host‐bone coverage. These technical constraints may have limited the adverse biomechanical effects of excessive superior placement and may partly account for the low rates of loosening and dislocation observed.

### 4.2 Long‐Term Hip Function and Patient‐Reported Health Status

Both groups showed substantial postoperative improvement in HHS. Compared with the true anatomic center group, the restricted quantitative superior center group showed significantly higher HHS values at 2, 7, and 8–10 years. SF‐36 scores improved in both groups but did not differ significantly between groups at any follow‐up point. One plausible explanation for the functional advantage in the restricted quantitative superior center group is the smaller magnitude of limb lengthening (5.56 vs. 11.85 mm), which may facilitate earlier and more symmetric postoperative gait. However, this remains hypothesis‐generating and should be examined in larger studies. These findings are broadly consistent with previous observational series: Murayama et al. reported postoperative HHS values of 88 and 82 for high and anatomic cup placement, respectively, after approximately 15 years [28]. A meta‐analysis of seven comparative studies likewise found no significant overall difference in postoperative HHS between the two techniques [29]. The discrepancy between these previous studies and our findings may partly reflect differences in study design and, importantly, heterogeneity in the definition and magnitude of superior displacement across studies.

### 4.3 Radiographic Outcomes and Acetabular Bone Grafting

#### 4.3.1 Hip‐Center Position

Since the early proposal of superior placement of the acetabular component at 35 mm proximal to the interteardrop line, studies have examined the safety and durability of the high hip center technique [24]. In our study, the acetabular component in the restricted quantitative superior center group was displaced by a mean of 9.15 mm above the true anatomic center, corresponding to a postoperative VD_cor_ of 31.66 mm. Fukui defined a high hip center as > 22 mm above the interteardrop line and reported that placement up to approximately 30 mm could achieve satisfactory outcomes [30], whereas Nawabi defined the high hip center as approximately 10 mm superior to the true anatomic center and demonstrated excellent survivorship and functional results [23]. Montalti et al. reported a mean center‐of‐rotation height of 33 mm [22], providing a useful radiographic comparison with the actual postoperative VD_cor_ observed in our restricted group (31.66 mm). In contrast, more pronounced superior placement, particularly > 35 mm above the interteardrop line, has been associated with increased prosthetic stress, polyethylene wear, loosening, dislocation, and impaired abductor function [31, 32]. Taken together, these findings suggest that the effect of superior placement may depend on the magnitude of displacement. Future large‐scale studies are needed to define the optimal range of superior displacement and to develop individualized reconstruction strategies based on patient‐specific acetabular anatomy and bone stock.

#### 4.3.2 Offset Restoration and Limb‐Length Management

The impact of superior cup placement on reducing abductor strength has been debated [33]. In the present study, postoperative FO and GFO were slightly greater in the restricted quantitative superior center group than in the true anatomic center group (FO, 34.32 vs. 31.15 mm; GFO, 61.97 vs. 58.55 mm), although neither difference reached statistical significance. Previous studies have shown that abductor strength is negatively correlated with the vertical distance of the hip, but positively correlated with FO [18, 19, 34]. Accordingly, when superior hip‐center placement is used, adjustment of FO through a longer prosthetic neck or modification of the neck‐shaft angle may help restore an appropriate abductor lever arm and preserve abductor strength [27, 35, 36, 37]. Considering that elderly patients often experience a decline in abductor strength, adjusting FO may become even more critical [38]. This indicates that surgeons may consider using a femoral prosthesis with high offset for balancing muscle strength when using superior placement, rather than choosing a larger femoral prosthesis.

Limb‐length management is another potential advantage of restricted quantitative superior placement. Although some studies have suggested that high hip center technique can reduce operative time and intraoperative blood loss, its effect on limb lengthening is less favorable [17]. In our study, postoperative LLD did not differ significantly between groups (3.26 vs. −1.90 mm), whereas limb lengthening was significantly smaller in the restricted superior group (5.56 vs. 11.85 mm). This may be attributed to appropriate selection of prosthetic neck length, optimized stem positioning by proper elevation of the stem. Moreover, in patients with bilateral DDH, restricted quantitative superior placement may improve limb symmetry and facilitate postoperative gait adaptation [39]. This finding was also consistent with our exploratory subgroup analysis of patients with bilateral DDH undergoing unilateral THA.

#### 4.3.3 Bone Coverage and Bone Grafting

Achieving at least 70% bone coverage of the acetabular cup is critical to ensure stability and promote adequate bone ingrowth [7, 10]. In our study, postoperative BCI was slightly higher in the restricted superior group than in the anatomic group (94.9% vs. 92.7%), with numerically lower rates of both morselized bone grafting (40.9% vs. 45.5%) and structural bone grafting (9.1% vs. 18.2%), although none of these differences reached statistical significance. Previous high hip‐center studies have suggested that superior placement may facilitate the use of more favorable host bone and reduce reliance on structural grafting [15, 23]. However, bone grafting remained necessary in some patients in our study, which may be related to the predefined 15‐mm limit on superior displacement and our relatively stringent requirement for BCI.

### 4.4 Limitations

First, the small sample size and 18.2% loss‐to‐follow‐up rate may have introduced attrition bias, reduced statistical power, and limited the feasibility of more detailed subgroup analyses. Second, the sample‐size calculation was based on historical reports available at the time of trial design. Because contemporary cementless anatomic acetabular reconstruction may achieve higher component survivorship than that reported in these earlier series, the survival rate of the control group may have been underestimated and the anticipated between‐group difference overestimated. Third, no revisions were recorded among the 36 hips that completed the 8–10‐year follow‐up, which precluded generation of prosthesis survivorship curves through Kaplan–Meier analysis. Finally, this was a single‐center trial. Future multicenter studies are warranted to validate the findings.

## 5 Conclusion

In selected patients with Crowe I–III developmental dysplasia of the hip, restricted quantitative superior placement within 15 mm above the true anatomic center was associated with higher HHS than true anatomic reconstruction at 8–10 years postoperatively. SF‐36 scores, bone‐grafting requirements, and prosthetic complications did not differ significantly between groups, and no revisions were recorded among the 36 hips that completed the 8–10‐year follow‐up. It may represent a potential reconstructive option for patients with DDH.

## Author Contributions

**Shuai Li:** conceptualization, methodology, data curation, writing – original draft, visualization, formal analysis, investigation, software. **Zhijian Yang:** conceptualization, writing – original draft, investigation, validation, funding acquisition. **Dong Jiang:** data curation. **Yu Xie:** data curation. **Xiaomin Lu:** data curation. **Xiaoyi Zhao:** data curation. **Minghui Gu:** data curation. **Zhiwen Li:** data curation. **Weiming Liao:** supervision, conceptualization, funding acquisition, writing – review and editing. **Zhiqi Zhang:** conceptualization, funding acquisition, project administration, resources, writing – review and editing.

## Funding

This study was supported by Sun Yat‐sen University 5010 Clinical Research Project (2013002), National Natural Science Foundation of China (82572796, 82172467), the First Affiliated Hospital of Sun Yat‐sen University Ke Ling Funding program for Novel and Distinguished talents (R07005), the Guangdong Natural Science Funds for Distinguished Young Scholar of China (2021B1515020008), Guangzhou Science Funds (2024A04J6487), and Guangdong Medical Research Foundation (A2023083).

## Ethics Statement

The study followed the Declaration of Helsinki. This study was approved by the Clinical Research Ethics Committee of The First Affiliated Hospital of Sun Yat‐sen University (IRB No. 2013‐032) on February 28, 2013. The trial was retrospectively registered at the Chinese Clinical Trial Registry on April 11, 2014 (registration number: ChiCTR‐TRC‐1404388).

## Conflicts of Interest

The authors declare no conflicts of interest.

## Supporting information


**Supplementary Table 1.** Prosthesis Manufacturers by Study Group.

**Supplementary Table 2.** Baseline characteristics of hips with and without complete follow‐up at 8–10 years.

**Supplementary Table 3.** Inter‐observer comparison.

**Data S1:** CONSORT 2025 checklist of information to include when reporting a randomized trial.

## Data Availability

The datasets used and/or analyzed during the current study are available from the corresponding author on reasonable request.
