# Supplementary material for: Long‐Term Efficacy of Restricted Quantitative Superior Placement Technique in Total Hip Arthroplasty for Severe Developmental Dysplasia of the Hip: An 8‐ to 10‐Year Follow‐Up of a Prospective Randomized Controlled Trial
Source: Orthop Surg. 2026 Nov 5:10.1111/os.70448. Online ahead of print. doi: 10.1111/os.70448 (PMC13739650; doi:10.1111/os.70448)
Supplement: Supplementary file 1 [file OS-9999-0-s001.docx]

**Supplement table 3.** Inter-observer comparison

| Radiographic parameter | Observer 1 | Observer 2 | ICC  (single measure) | 95% CI | Statistics | *P* |
| --- | --- | --- | --- | --- | --- | --- |
| Preoperative LLD (mm) | 9.53±15.06 | 9.24±15.26 | 0.997 | 0.995-0.998 | 668.680 | **<0.001** |
| Preoperative VD*_cor_* (mm) | 22.36±1.49 | 22.55±1.34 | 0.840 | 0.725-0.909 | 6.342 | **<0.001** |
| Sharp angle (deg) | 47.05±4.98 | 46.86±5.40 | 0.959 | 0.927-0.978 | 48.142 | **<0.001** |
| Acetabular depth-to-width ratio(%) | 25.63±4.92 | 25.55±5.02 | 0.971 | 0.947-0.984 | 67.082 | **<0.001** |
| Postoperative radiographic parameters | | | | | | |
| Superior placement distance (mm) | 4.71±5.52 | 4.30±5.79 | 0.961 | 0.929-0.978 | 49.677 | **<0.001** |
| VD*_cor_* (mm) | 27.45±5.87 | 26.48±5.82 | 0.981 | 0.963–0.990 | 104.758 | **<0.001** |
| VDgt (mm) | 25.53±9.38 | 25.43±9.16 | 0.956 | 0.920-0.975 | 43.991 | **<0.001** |
| FO (mm) | 32.98±6.13 | 32.49±5.87 | 0.937 | 0.888-0.965 | 30.918 | **<0.001** |
| GFO (mm) | 60.40±6.71 | 60.12±6.46 | 0.931 | 0.876-0.962 | 27.803 | **<0.001** |
| LLD (mm) | 0.89±14.06 | 0.48±14.40 | 0.986 | 0.975-0.993 | 145.333 | **<0.001** |
| BCI (%) | 93.94±5.61 | 93.63±6.12 | 0.866 | 0.768-0.925 | 13.966 | **<0.001** |
| Cup anteversion (deg) | 24.05±9.35 | 23.56±10.14 | 0.960 | 0.927-0.978 | 48.580 | **<0.001** |
| Cup inclination (deg) | 45.49±7.82 | 44.77±7.78 | 0.956 | 0.920-0.976 | 44.255 | **<0.001** |

Values for Observer 1 and Observer 2 are presented as mean ± standard deviation. ICCs and their 95% confidence intervals describe the reliability of individual measurements between the two observers.. Bold values indicate statistically significant differences (*P* < 0.05). ICC, intraclass correlation coefficient; CI, confidence interval; SD, standard deviation; VD*_cor_*, vertical distance from center of rotation to interteardrop line; VD*_gt_*, vertical distance from tip of greater trochanter to interteardrop line; FO, femoral offset. GFO, global femoral offset; LLD, leg length discrepancy; BCI, bone coverage index.
