# Supplementary material for: Long‐Term Efficacy of Restricted Quantitative Superior Placement Technique in Total Hip Arthroplasty for Severe Developmental Dysplasia of the Hip: An 8‐ to 10‐Year Follow‐Up of a Prospective Randomized Controlled Trial
Source: Orthop Surg. 2026 Nov 5:10.1111/os.70448. Online ahead of print. doi: 10.1111/os.70448 (PMC13739650; doi:10.1111/os.70448)
Supplement: Supplementary file 2 [file OS-9999-0-s002.docx]

|  | True anatomic  center (n=22) | Restricted quantitative superior center (n=22) |
| --- | --- | --- |
| DePuy | 15（68.2%） | 15（68.2%） |
| Smith & Nephew | 3（13.6%） | 2（9.1%） |
| Biomet | 4（18.2%） | 5（22.7%） |

**Supplementary Table 1.** Prosthesis Manufacturers by Study Group
