# Supplementary material for: Long‐Term Efficacy of Restricted Quantitative Superior Placement Technique in Total Hip Arthroplasty for Severe Developmental Dysplasia of the Hip: An 8‐ to 10‐Year Follow‐Up of a Prospective Randomized Controlled Trial
Source: Orthop Surg. 2026 Nov 5:10.1111/os.70448. Online ahead of print. doi: 10.1111/os.70448 (PMC13739650; doi:10.1111/os.70448)
Supplement: Supplementary file 4 [file OS-9999-0-s004.docx]

**Supplement Table 2.** Baseline characteristics of hips with and without complete follow-up at 8–10 years

|  | Completed follow-up (n=36) | Lost to follow-up  (n=8) | Statistics | *P* |
| --- | --- | --- | --- | --- |
| Age (years) | 57.78 ± 10.34 | 60.88 ± 6.58 | -0.808 | 0.424 |
| Height (cm) | 158.92 ± 7.82 | 160.38 ± 3.82 | -0.778 | 0.445 |
| Weight (kg) | 62.58 ± 12.75 | 59.13 ± 12.41 | 0.697 | 0.490 |
| BMI (kg/m^2^) | 24.77 ± 4.74 | 22.92 ± 4.29 | 1.082 | 0.302 |
| Sex, no. of hips (%)  Female  Male | 27 (75.0)  9 (25.0) | 3 (37.5)  5 (62.5) |  | 0.087 |
| Side for THA, no. of hips (%)  Left  Right | 17 (47.2)  19 (52.8) | 6 (75.0)  2 (25.0) |  | 0.245 |
| Average subluxation (%) | 47.21 ± 13.37 | 53.42 ± 17.26 | -1.134 | 0.263 |
| Preoperative HHS | 50.35 ± 11.41 | 50.56 ± 6.69 | -0.070 | 0.945 |
| Preoperation SF-36 | 59.78 ± 6.53 | 61.13 ± 8.59 | -0.499 | 0.621 |
| Preoperative LLD (mm) | 9.45 ± 15.19 | 9.10 ± 16.01 | 0.058 | 0.954 |
| Preoperative VD*_cor_* (mm) | 22.45 ±1.32 | 22.48 ± 1.59 | -0.054 | 0.957 |
| Sharp angle (deg) | 46.97 ±5.02 | 47.37±5.12 | -0.202 | 0.839 |
| Acetabular depth-  to-width ratio(%) | 24.98±4.69 | 28.34±5.40 | -1.789 | 0.081 |

Continuous variables are reported as mean ± standard deviation. Categorical variables are presented as number (%). Comparisons between groups were performed using *t* test or Mann-Whitney *U* test, as appropriate. Chi-squared (χ²) test is used to compare categorical variables. THA, total hip arthroplasty; BMI, body mass index; LLD, limb length discrepancy; HHS, Harris Hip Score; VD*_cor_*, vertical distance from center of rotation to interteardrop line.
